# Supplementary figures and images for: Missense variants causing Wiedemann-Steiner syndrome preferentially occur in the KMT2A-CXXC domain and are accurately classified using AlphaFold2
Source: PLoS Genet. 2022 Jun 21;18(6):e1010278. doi: 10.1371/journal.pgen.1010278 (PMC9249231; doi:10.1371/journal.pgen.1010278)

A

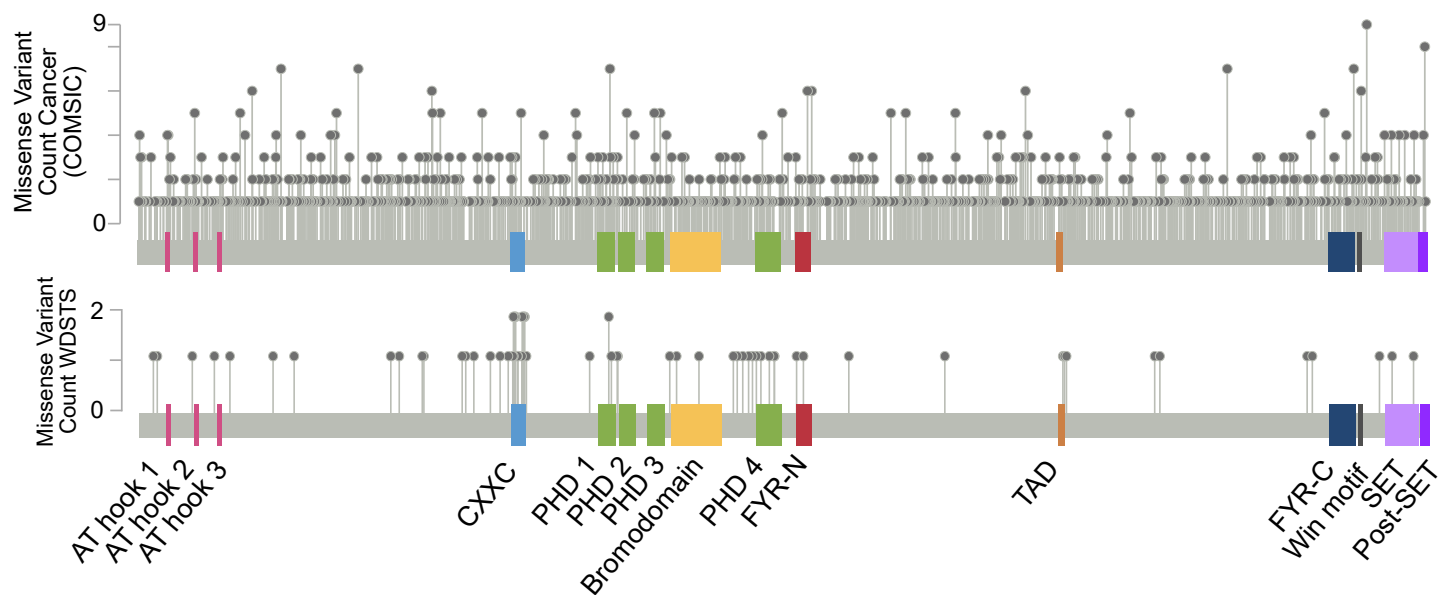

B

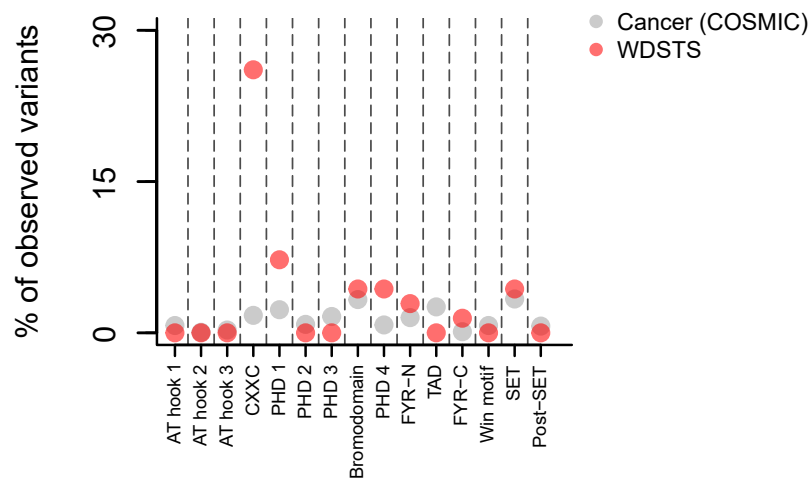

C

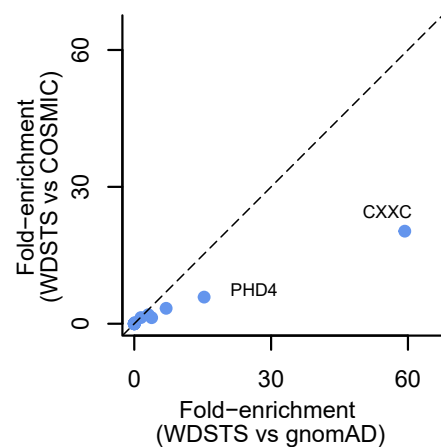

D

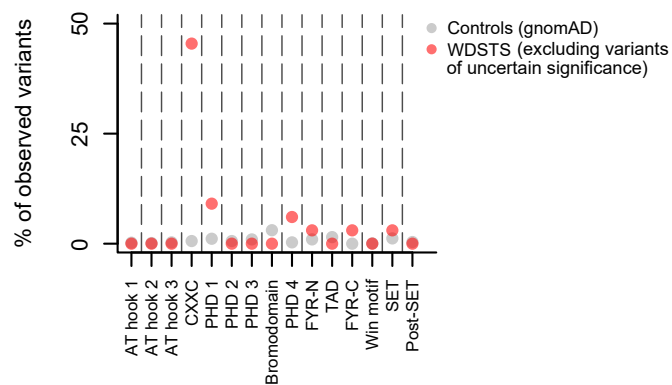

E

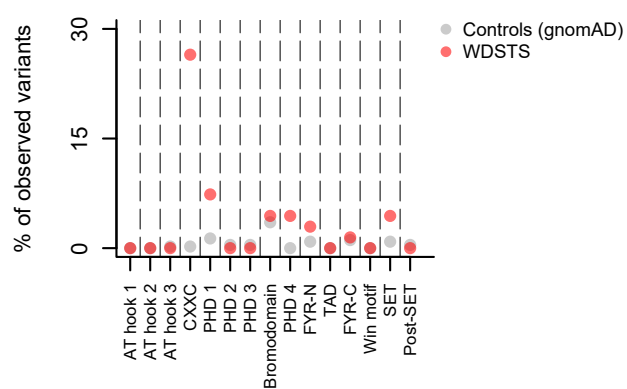

Supplement: S1 Fig — (A) KMT2A missense variants in COSMIC (top) and ClinVar (bottom). (B) The percentage of missense variants in COSMIC (grey dots) and WDSTS patients (red dots) that fall in each of the different domains of KMT2A. (C) Correlation between the fold-enrichment (odds ratio) of WDSTS MVs compared to COSMIC MVs and gnomAD MVs. (D) The percentage of missense variants in gnomAD (grey dots) and WDSTS patients (red dots) that fall in each of the different domains of KMT2A, after excluding variants of uncertain significance. (E) The percentage of missense variants in gnomAD (grey dots) and WDSTS patients (red dots) that fall in each of the different domains of KMT2A, after excluding gnomAD variants with MAF<10e-5. (PDF) [file pgen.1010278.s001.pdf]

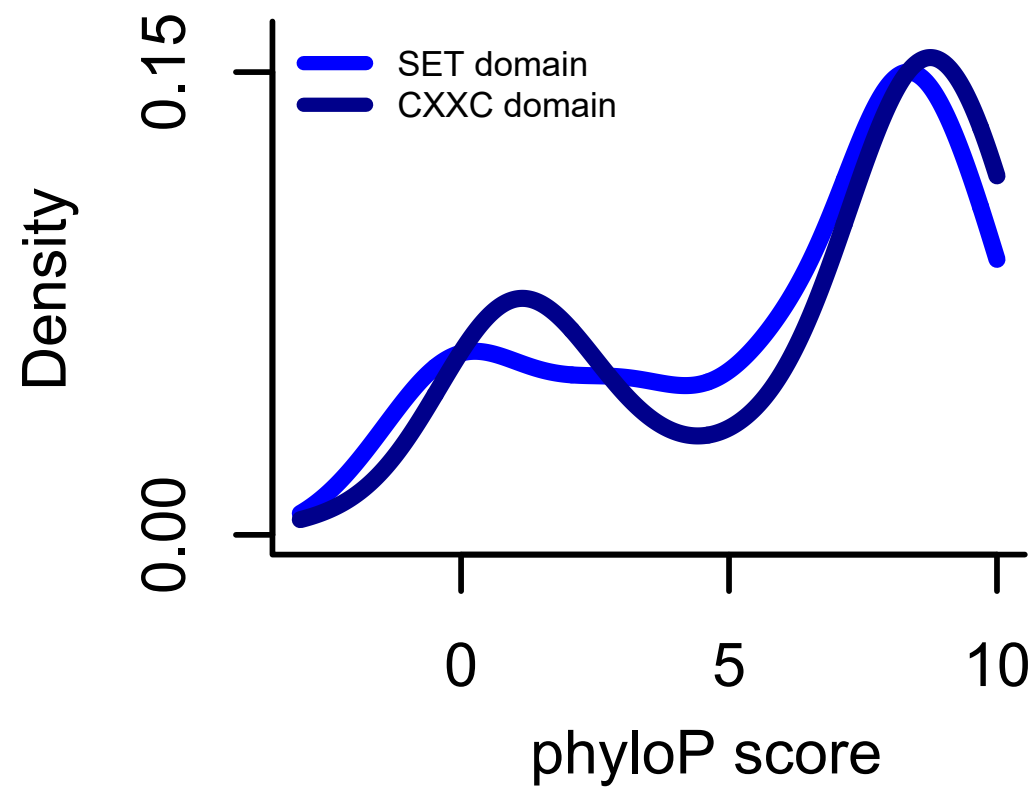

Supplement: S2 Fig — (PDF) [file pgen.1010278.s002.pdf]

A

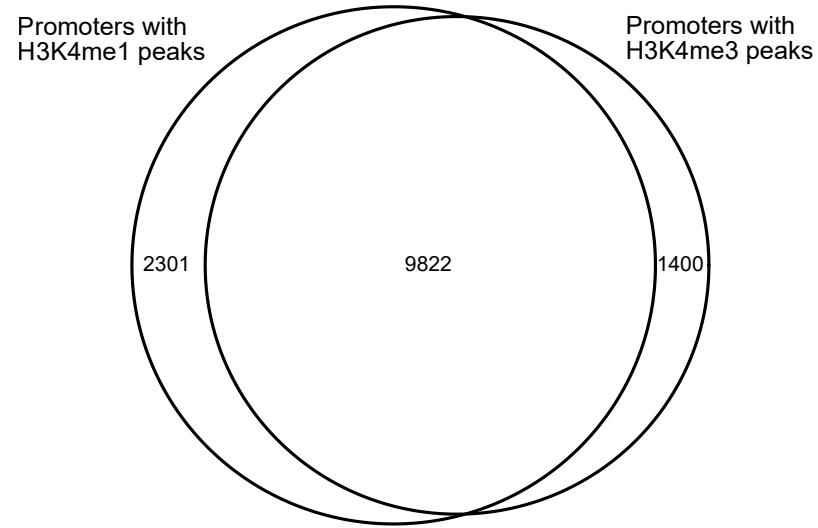

B

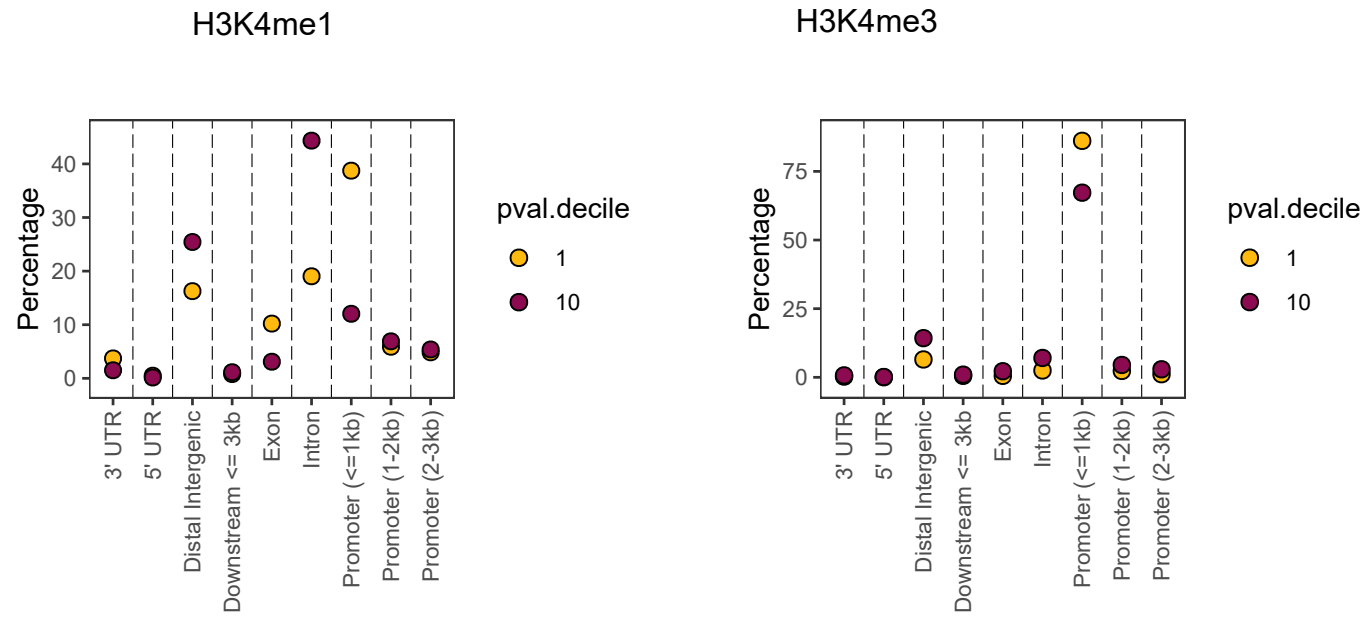

Supplement: S3 Fig — (A) Venn diagram depicting the overlap between promoters (+/- 1kb from TSS) harboring H3K4me1 peaks and those harboring H3K4me3 peaks. (B) Genomic annotation of peaks within the 1st (yellow dots) and 10th p-value decile (purple dots) for H3K4me1 peaks (left) and H3K4me3 peaks (right). (PDF) [file pgen.1010278.s003.pdf]

A

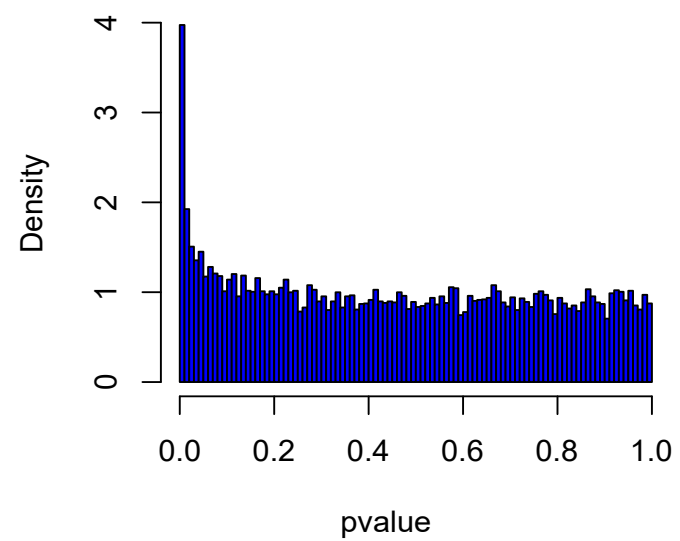

B

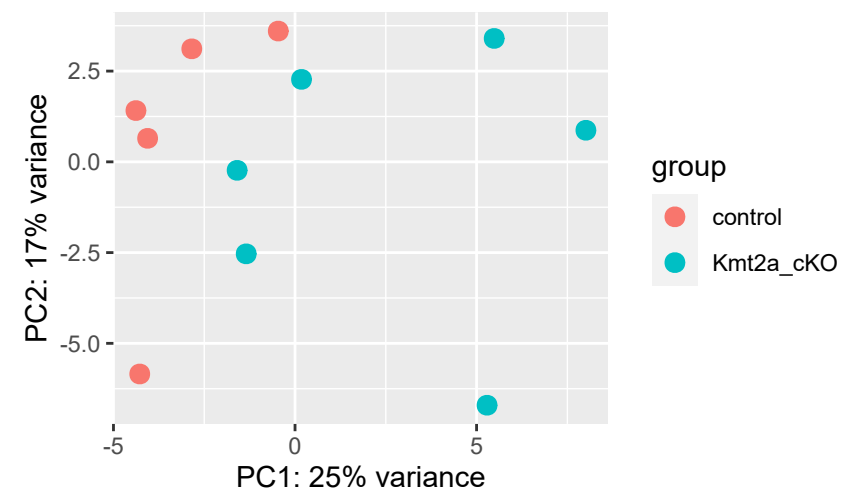

C

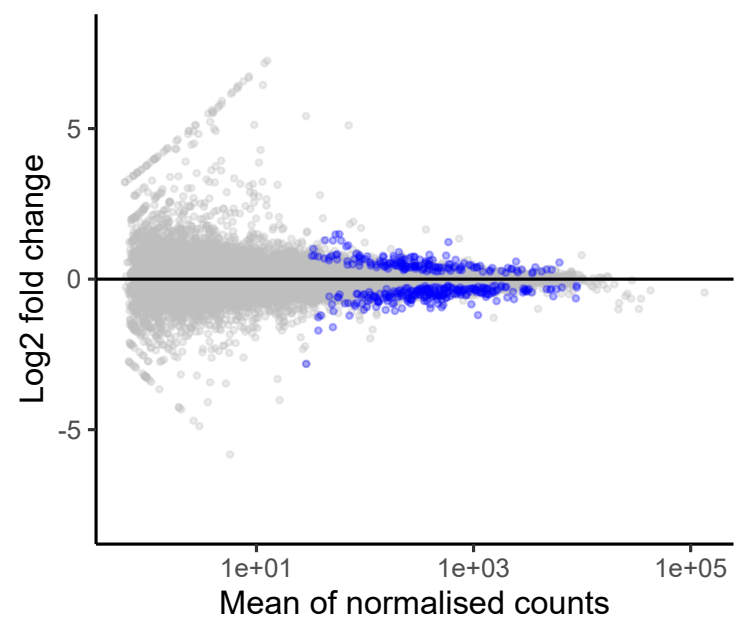

Supplement: S4 Fig — (A) The histogram of p-values from the differential expression RNA-seq analysis. (B) PCA plot based on the expression matrix, after a variance stabilizing transformation (see Methods). (C) MA plot of the log2 fold-change against the mean of normalized counts from the differential expression analysis. Differentially expressed genes are colored in blue. (PDF) [file pgen.1010278.s004.pdf]

A

Per-Residue Count of Non-Gap Amino Acids in the MSA

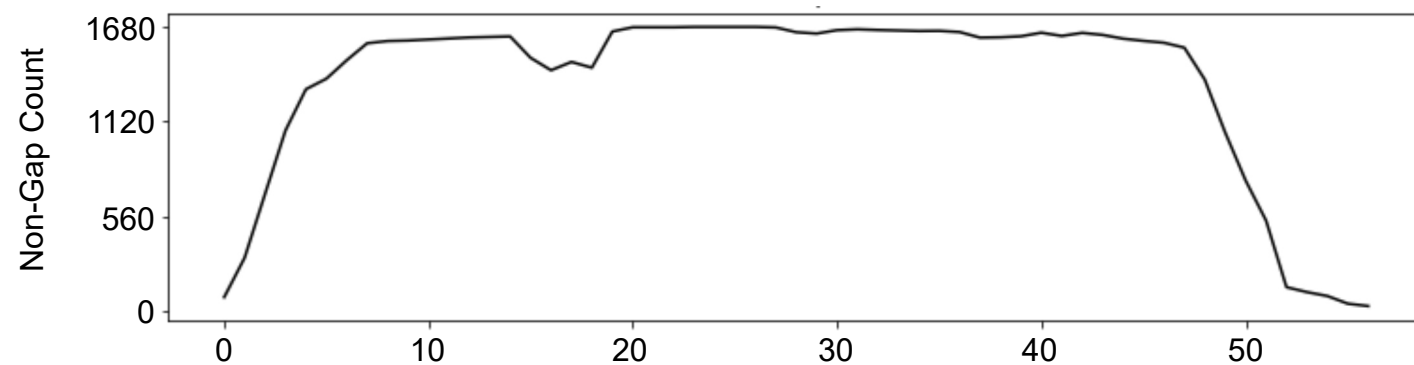

B

Predicted Aligned Error

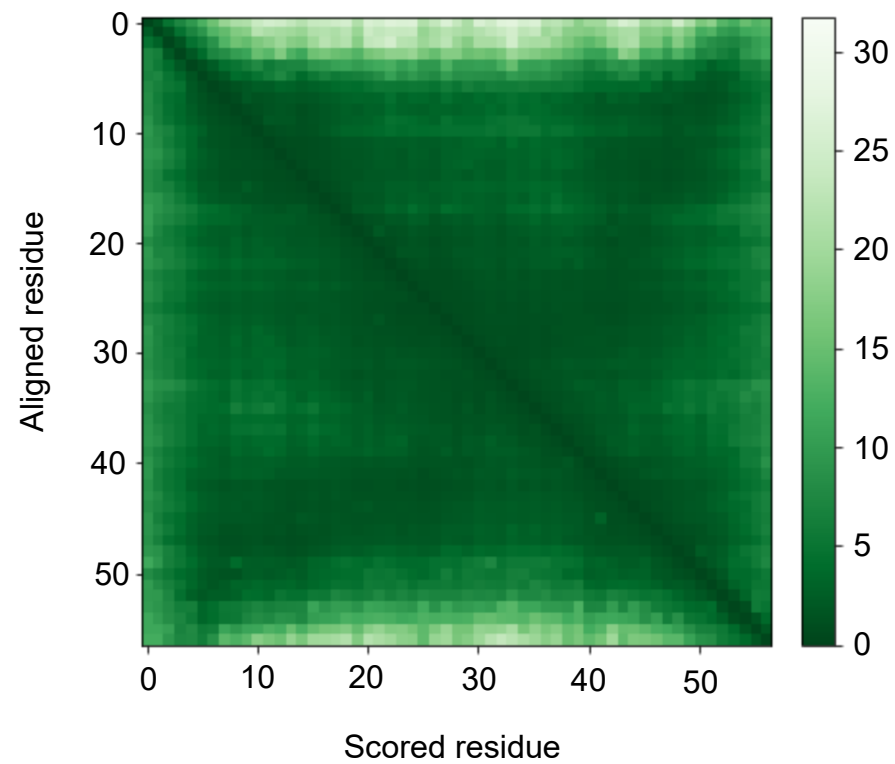

Supplement: S5 Fig — (A) Multiple sequence alignment depth plot and (B) predicted alignment error of KMT2A CXXC wild-type domain prediction from AlphaFold2. (PDF) [file pgen.1010278.s005.pdf]

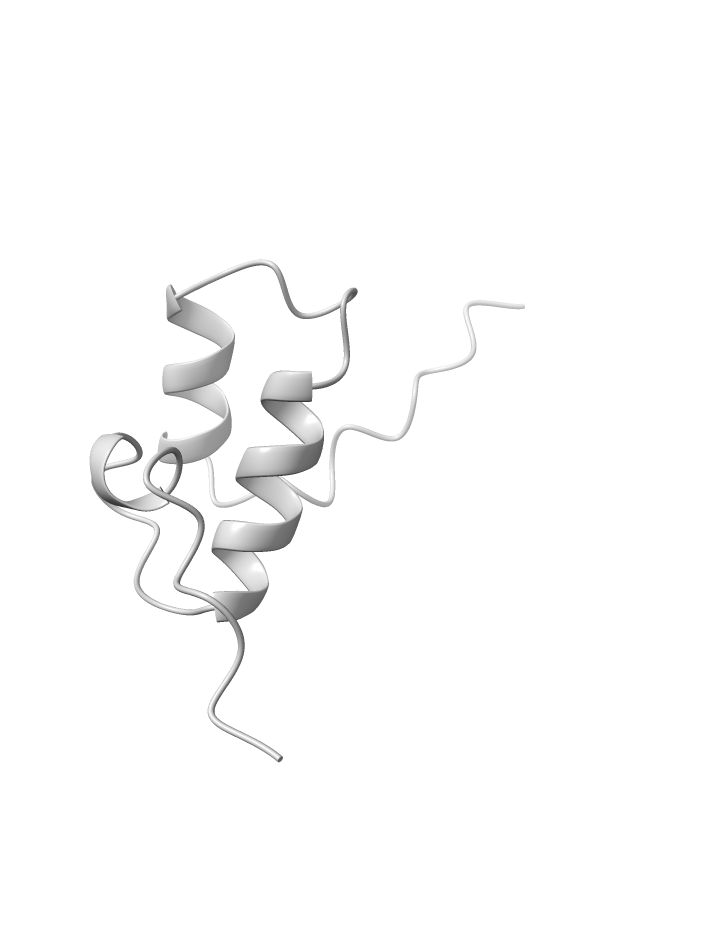

Supplement: S6 Fig — Variant classification scheme from the (A) training set and (B) test set. Variants that do not fall under the correct classification according to the scheme are underlined. (C) Confusion matrix from test set results. (PNG) [file pgen.1010278.s006.png]

A

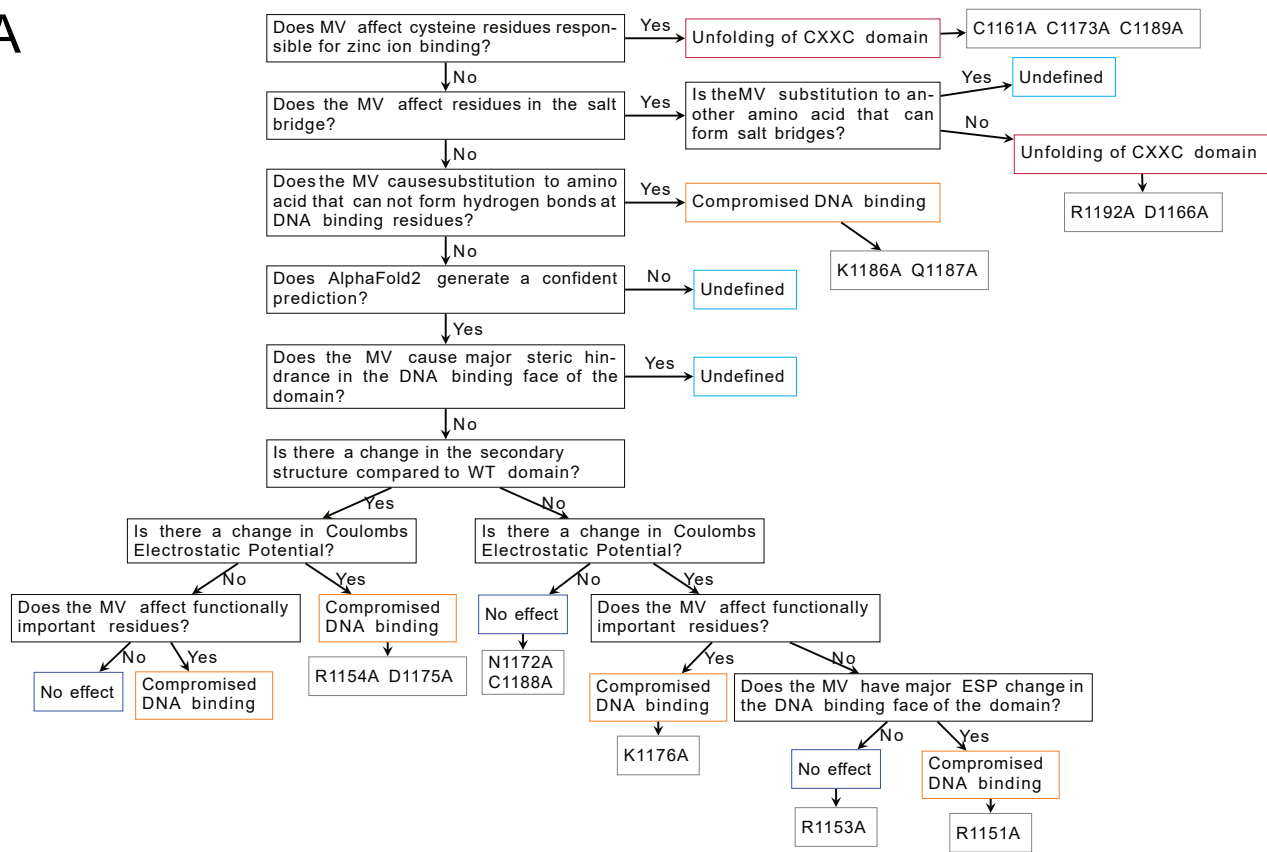

B

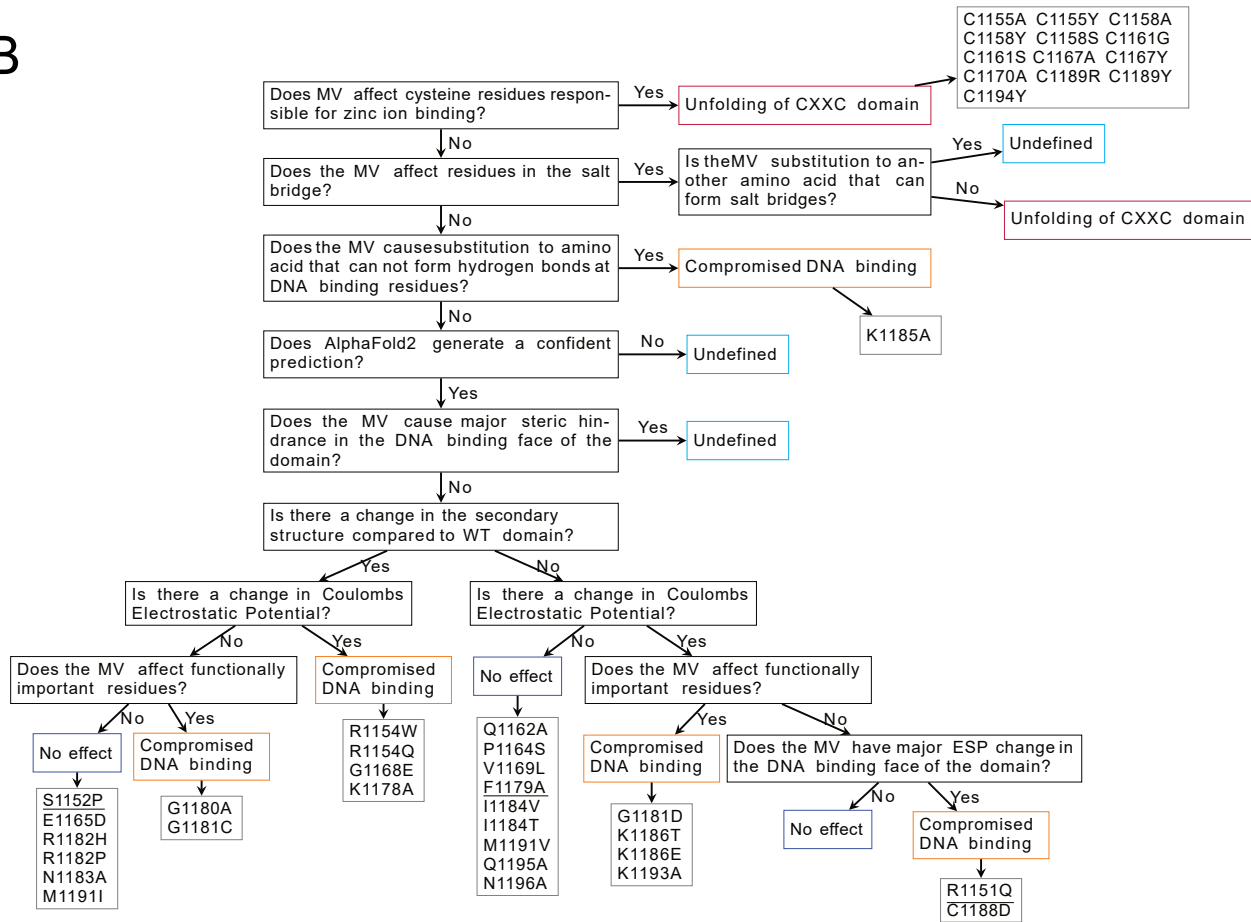

C

|      |          | Predicted |          |       |
|------|----------|-----------|----------|-------|
|      |          | benign    | damaging | total |
| True | benign   | 25        | 1        | 26    |
|      | damaging | 2         | 13       | 15    |
|      | total    | 27        | 14       |       |

Supplement: S7 Fig — (PDF) [file pgen.1010278.s007.pdf]
