# Supplementary material for: Missense variants causing Wiedemann-Steiner syndrome preferentially occur in the KMT2A-CXXC domain and are accurately classified using AlphaFold2
Source: PLoS Genet. 2022 Jun 21;18(6):e1010278. doi: 10.1371/journal.pgen.1010278 (PMC9249231; doi:10.1371/journal.pgen.1010278)
Supplement: S2 Table — (PDF) [file pgen.1010278.s009.pdf]

Functionally important residues in the CXXC domain of KMT2A

Residues implicated in electrostatic interaction with the DNA	Arg1150, Arg1154, Lys1176, Lys1178, , Lys1190, Arg1192, Lys1193
Residues responsible for hydrogen bond formation with the DNA	Lys1185, Lys1186, Gln1187
Residues at the KFGG site	Lys1178, Phe1179, Gly1180, Gly1181
