# Supplementary material for: Missense variants causing Wiedemann-Steiner syndrome preferentially occur in the KMT2A-CXXC domain and are accurately classified using AlphaFold2
Source: PLoS Genet. 2022 Jun 21;18(6):e1010278. doi: 10.1371/journal.pgen.1010278 (PMC9249231; doi:10.1371/journal.pgen.1010278)
Supplement: S5 Table — (PDF) [file pgen.1010278.s012.pdf]

**Domain**	**Amino acid co-ordintes** ENSP00000436786.1
AT hook 1	169-180
AT hook 2	217-227
AT hook 3	301-309
CXXC Domain	1147-1203
PHD finger 1	1433-1480
PHD finger 2	1481-1531
PHD finger 3	1568-1625
Bromodomain	1636-1770
PHD finger 4	1935-1981
FYR-N Domain	2021-2077
TAD Domain	2850-2858
FYR-C Domain	3669-3756
Win Motif	3765-3773
SET Domain	3832-3954
Post-SET Domain	3956-3972
