# Supplementary material for: Missense variants causing Wiedemann-Steiner syndrome preferentially occur in the KMT2A-CXXC domain and are accurately classified using AlphaFold2
Source: PLoS Genet. 2022 Jun 21;18(6):e1010278. doi: 10.1371/journal.pgen.1010278 (PMC9249231; doi:10.1371/journal.pgen.1010278)
Supplement: Attachment — Submitted filename: PLOS_Responses_.docx [file pgen.1010278.s013.docx]

Missense variants causing Wiedemann-Steiner syndrome preferentially occur in the KMT2A-CXXC domain and are accurately classified using AlphaFold2

(PGENETICS-D-22-00064)

Response to reviewers

Tinna Reynisdottir, Kimberley Jade Anderson, Leandros Boukas, Hans Tomas Bjornsson

We thank the reviewers for the careful reading of our manuscript and their thoughtful comments, which have led to an improved manuscript. The reviewers’ comments have led to the addition of 7 main figure panels, 3 supplemental figures, and significant modification of our text.

Reviewer 1

The work is well designed and it gives evidence from different viewpoints on the relevance of the CXXC domain on the Wiedemann Steiner Syndrome (WSS) pathogenesis. However, it needs some further explanations in order to be published.

**Response**: We thank Dr. Faundes for his kind words about our work.

Major comments:

The use of COSMIC variants as evidence of the enrichment of missense variants (MV) should be used cautiously. As authors may know, the translocation of KMT2A, which is part of the pathogenesis of certain leukemias, causes a kind of gain-of-function in KMT2A. It is likely some of these somatic MV may have a similar mechanism, and therefore, it is not applicable to WSS mechanism.

**Response:** We have now acknowledged the possibility that some of the COSMIC MVs may act via gain-of-function mechanisms, and we explicitly state that the enrichment of these MVs at different domains should be interpreted with caution when it comes to suggesting tumorigenic mechanisms. We now write in our results:

*“To assess the robustness of our result, we repeated our analysis using a control set of 1788 KMT2A somatic variants obtained from sequencing of tumor samples (****Methods****). While these variants are likely a mix of driver and passenger variants, the latter are expected to comprise the majority, justifying the use of this set of variants as controls alternative to gnomAD. We recapitulated our result, with the rank ordering of the different domains with respect to their enrichment of WDSTS MVs remaining unchanged (****Supplemental Figure 1A, B, C****); this is consistent with the pool of somatic variants containing mostly benign passenger variants. However, across all domains, the enrichment estimates are attenuated compared to the gnomAD-vs-WDSTS comparison (****Supplemental Figure 1C)****. This suggests that the same domains that contribute to WDSTS pathogenesis contribute to the tumorigenic role of KMT2A as well. With respect to this tumorigenic role, however, our result should be interpreted with caution; gain-of-function mechanisms that are likely not captured by our analysis are probably also involved, as evidenced from KMT2A translocations that act as drivers in certain types of leukemias.”*

Albeit ClinVar is a good source of MVs detected in clinical settings, few variants have been extensively curated in terms of interpretation. Considering this, the exclusion of Human Gene Mutation Database (HGMD) as source of confident pathogenic variants must be explained.

**Response:** We have now also included variants from the HGMD. There are total of 59 WDSTS variants in HGMD; 46 of these we had already obtained via ClinVar or WDSTS literature, whereas 2 have a phred-like CADD score below 20 (which is our threshold for inclusion). This leaves us with 11 new variants, which we have now used in our analyses. This has increased the power of our domain enrichment analysis (see also response to a comment below). Our main result remains unaltered: there is a 61.9-fold enrichment of missense variants within the CXXC domain. In addition, two of these newly added variants fall within the CXXC domain, and therefore we also included them in our hold-out test set that we use to evaluate the performance of our AlphaFold2-based classifier; both are classified correctly. We have updated our manuscript, figures, and supplemental data accordingly, and we now write in our methods:

*“In the case of WDSTS, we obtained 11 additional variants from the Human Gene Mutation Database (HGMD) with CADD score above 20, as well as 16 MVs from Lebrun et al, Baer et al, Miyake et al, WD Jones (all studies of individuals with a WDSTS clinical phenotype;* ***Supplemental Table 1)*** *[1-5].”*

***“Figure 1.*** *The distribution of likely pathogenic Wiedemann-Steiner syndrome missense variants across the different domains of KMT2A. (****A****) KMT2A missense variants in gnomAD (top) and WDSTS (bottom). See Methods for filtering criteria. (****B****) The percentage of missense variants from gnomAD (grey dots) and WDSTS (red dots) that fall in each of the different domains of KMT2A. (****C****) The percentage of missense variants in gnomAD (grey dots) and likely pathogenic variants (blue dots) that fall in the CXXC domain of different epigenetic regulators. (****D****) Multiple sequence alignment of the amino-acid sequence of the CXXC domain of KMT2A in eight eukaryotic species. Residues known to be important for DNA binding are marked with red asterisks at the top (see Methods for details). The eight zinc ion-binding cysteines are marked with red asterisks at the bottom.”*

It is seems a bit confusing the inclusion and exclusion of VUSes in the initial analyses. It seems logical to use them when AlphaFold is applied for reinterpretation, not for generating the evidence of the relevance of CXXC domain in WSS.

**Response:** The choice of including VUS’s or not inevitably comes down to a trade-off between power and signal-to-noise ratio. By including VUS’s we are increasing our power because we have a greater number of variants (some of which are truly pathogenic). But this comes at the expense of a lower signal-to-noise ratio, since some of the VUS variants are not pathogenic. By contrast, when excluding VUS’s we are increasing our signal-to-noise ratio, as we ensure that we only use variants that are truly pathogenic; however, we have less power, since power depends critically on the number of variants used. Initially, we opted for more power, which is why we included VUS’s. However, prompted by Dr. Faundes’ comment, and given that we now also include 11 new variants from HGMD (see response to comment above), we have now conducted an analysis where we exclude all of the VUS’s and only use variants of known pathogenicity (new supplemental Figure 1D; shown below). This analysis recapitulates the results obtained from our initial analysis that includes VUS’s: the CXXC domain shows by far the greatest enrichment of WDSTS missense variants, with PHD fingers 1 and 4 being next. In addition, the resulting enrichment estimates are more pronounced than these obtained with the VUS’s, but the confidence intervals are wider, consistent with the notion that we have increased signal-to-noise ratio but less power. We now write in our Methods:

*“To ensure that our domain enrichment estimates are not artifacts driven by the inclusion of VUS’s, we also performed the domain enrichment analysis after excluding VUS’s and obtained very similar results* ***(Supplemental Figure 1D).*** *Without VUS’s, the enrichment in the CXXC domain is even greater, but the confidence interval around the point estimate is wider (Supplemental Figure 1D; confidence interval = 49.2 - 442.8 without VUS’s vs 24.3 - 173.4 when including VUS’s). The same is true for the 1st and 4th PHD finger, which again show significant enrichment as well (Supplemental Figure 1D; For PHD finger 4; confidence interval = 1.9 - 163.0 without VUS’s vs 2.3 - 97.0 when including VUS’s and for PHD finger 1; confidence interval = 1.6 - 35.0 without VUS’s vs 1.9 - 20.4 when including VUS’s). Together, these results are consistent with the notion that, by excluding VUS’s, we are increasing our signal-to-noise ratio, since we are not including any variants with uncertain pathogenicity. However, this comes at the expense of less power (reflected in the greater uncertainty around the enrichment point estimates), since this analysis is inevitably conducted using fewer variants.”*

***“Supplemental Figure 1D:*** *The percentage of missense variants in gnomAD (grey dots) and WDSTS patients (red dots) that fall in each of the different domains of KMT2A, after excluding variants of uncertain significance.”*

Considering recent evidence of the incidence of WSS in newborns (~1/8600 according to Brain. 2020 Apr; 143(4): 1099–1105) and the cutoffs proposed by Whiffin et al. 2017, the MAF cutoff for controls seems too low. Also, it has been demonstrated that genes involved in epigenetic machinery (like KMT2A) are susceptible to clonal hematopoiesis of indeterminate potential (CHIP) and therefore, very-rare control MV may still be pathogenic. These facts are especially crucial to consider, especially when SET domain was found not to be enriched in this work, which may change if another cutoff is considered.

**Response:** First, we would like to point out that while in the original submission we indicated that we imposed a MAF cutoff on the gnomAD variants, that was an error in the Methods section. While we did experiment with different cutoffs, we ended up including all gnomAD variants. This is because most of them are very rare and were being excluded when applying the cutoffs, leaving us with very few control variants to conduct the analysis (e.g. 1340 out of 1403 gnomAD variants have a MAF less than 0.0001). Nevertheless, we fully agree that it is important to check that the result doesn’t change if a cutoff is applied. To this end, we have now performed a domain enrichment analysis solely with gnomAD variants with MAF>10e-5 (452 variants). Reassuringly, the results are very similar; the CXXC domain still shows the greatest enrichment (odds ratio = 159.4, Fisher’s exact test, p = 3.66e-15), and the SET domain shows no significant enrichment (Figure shown below). It is also worth mentioning here that the fact that we obtain similar enrichment estimates for the different domains when we use the COSMIC variants as controls is also reassuring, since these somatic variants are subject to very different selective pressures than the germline variants contained in gnomAD.

We now write in Methods:

*“Finally, since it is possible that very rare MVs in gnomAD may in fact be pathogenic, we assessed whether our result still holds when excluding low-frequency variants, and found this to be true. Using only variants with MAF great than 10e-5 (452 variants), we found that the CXXC domain still shows the greatest enrichment (odds ratio = 159.4, Fisher’s exact test, p = 3.66e-15), followed by PHD fingers 4 and 1 (for PHD finger 4; odds ratio = inf, Fisher’s exact test, p = 0.0322 and for PHD finger 1; odds ratio = 5.8, Fisher’s exact test, p = 0.122), while the SET domain shows no significant enrichment (Fisher’s exact test, p = 0.762),* ***Supplementary Figure 1E****. “*

***“Supplemental Figure 1E:*** *The percentage of missense variants in gnomAD (grey dots) and WDSTS patients (red dots) that fall in each of the different domains of KMT2A, after excluding gnomAD variants with MAF<10e-5.”*

Although the AlphaFold predicted the best structure of CXXC domain, it would be very graphical and reader-friendly to depict some pathogenic MV detected in silico into the 4nw3 structure, and how they affect the interaction with DNA (which was also determined in that structure).

**Response:** We thank Dr. Faundes for this suggestion, and we agree that such a visual representation is helpful and intuitive. We have now included a new panel in figure 3 (shown below) which shows the position of two different missense variants in the 3D structure of the CXXC domain, in conjunction with a 3D representation of the engaged DNA backbone. We use color coding to show that both of these variants cause a decrease in the electrostatic potential of the domain, which sheds light on their pathogenicity as it is known that DNA binding depends on the appropriate electrostatic interactions between the CXXC domain and the DNA.

*“As an example, in* ***Figure 3E*** *we depict two WDSTS pathogenic variants which our classifier correctly labels as compromising DNA binding because they decrease the electrostatic potential at residues important for the formation of contacts with the DNA backbone. Notably, out of the 18 WDSTS MVs present in the CXXC domain of KMT2A, nine are positioned at cysteine residues responsible for zinc ion binding compared to none of the control variants (Fisher’s exact test, p = 0.009816).”*

Figure 3 is now:

***“Figure 3. An AlphaFold2-based variant effect classification scheme for the CXXC domain of KMT2A.*** ***(A)*** *Predicted LDDT values for the CXXC domain of KMT2A.* ***(B)*** *The AlphaFold2-predicted and experimentally determined structures of the CXXC domain of KMT2A.* ***(C)*** *The variant effect classification scheme. See* ***Methods*** *for details on the derivation of the scheme.* ***(D)*** *The positive and negative predictive value that the classifier shown in* ***(C)*** *attained on a hold-out test set consisting of 41 missense variants (see main text and* ***Methods*** *for details).* ***(E)*** *The position of two different missense variants in the 3D structure of the CXXC domain, in conjunction with a 3D representation of the engaged DNA backbone. For comparison, the same representation is shown for the normal protein as well (PBD ID: 4NW3). The surface of the domain is color-coded based on the electrostatic potential, with red indicating a negative charge and blue a positive charge. Both variants lead to a decreased electrostatic potential in a residue important for DNA binding.”*

Minor Comments:

Please in supp table S4 describe the exact variants following the HGVS nomenclature and their two interpretations (pre- and post- AlphaFold analysis).

**Response:** We have now added the variant transcript IDs according to the HGVS nomenclature in supplemental table 4. We also added two columns to supplemental table 3, where we list all variants in our training and test set, with the additional information suggested by the reviewer. We also describe each variant used along with their two interpretations: their determined effect (pre - AlphaFold) and their variant classification result (post - AlphaFold).

In the Methods section, "Missense variants" subsection, write the genes involved in each of the phenotypes "(Wiedemann-Steiner syndrome, Hereditary sensory 221 neuropathy type 1E, Childhood-onset dystonia, and Beck-Fahrner syndrome)"

**Response:** We have now included the causative genes for each of the disorders. We now write in Methods:

*“MVs present in individuals with disease phenotypes (Wiedemann-Steiner syndrome [KMT2A], Hereditary sensory neuropathy type 1E [DNMT1], Childhood-onset dystonia [KMT2B], and Beck-Fahrner syndrome [TET3]) were obtained from ClinVar [6].”*

In Figure 3B, please mention the pdb codes of the different structures.

**Response:** We have now done this.

Reviewer 2

This study examined variants in KMT2A related to Weidemann-Steiner syndrome to seek insight in to the pathogenesis of the syndrome. First, they identified the locations of pathogenic variants in KMT2A and found them overrepresented in the CXXC domain. This pattern of variant locations was found to be unique to KMT2A, as compared to three other genes which cause Mendelian diseases and did not have pathogenic variants overrepresented in their CXXC domains. To identity the biological impact of KMT2A mutations the authors analyzed previously published data to determine the effect of KMT2A loss on H3K4me3 binding (ChIP-seq) and gene expression (RNA-seq) in WT and KMT2A conditional KO mouse hippocampal neurons. They found that, while H3K4me3 was most disrupted in CpG-rich regions (matching the known function of KMT2A of binding unmethylated CpGs), including at promoters, the expression of nearby genes was not affected by the H3K4me3 loss. Lastly, they used the AlphaFold2 software in conjunction with variants with experimentally verified or in silico predicted pathogenicity to develop an AlphaFold2-based variant classification system with over 90% accuracy, then applied it to most variants in the CXXC domain of KMT2A.

This study has clear formulations of its goals: how important is the role of the different KMT2A domains in WSS pathogenesis and what are rules that determine how the variants – genetic disruption – in these domains causes WSS. To achieve the stated goals it uses simple, quite clear albeit novel methodology in the second goal by linking a genetic change to the protein function through the in-silico modeling of the protein folding. By comparing disease causing variant fraction to the control non-pathogenic variant fraction in the KMT2A domains the study identifies the CXXC domain as having the most significant role among other domains in WSS pathogenesis. Using KMT2A-deficient mice to show that H3K4me1 disturbances preferentially occur at CpG-rich regions, but has no systematic effect on gene expression (via ChIP and RNA-seq)
**Response:** We thank the reviewer for their kind words about our study.

Major Weaknesses

It is recommended to characterize the conservation properties of the domain across species in the orthologous genes. That would provide stronger evidence of the importance of CXXC domain and its intolerance to perturbations. This is missing in the study as well as the genomic coordinates and identities of the transcripts (or a single major transcript) for which the missense variants were collected.

**Response:** We have now examined the conservation of the CXXC domain, by performing the following analyses. First, we did an alignment of the amino-acid sequences of the domain from 8 eukaryotic species (**Figure 1D**). Even though these species span a very wide range of eukaryotic diversity, the conservation of the domain is evident, especially at the most important residues (indicated on the alignment). Second, for a more comprehensive analysis of the conservation of the CXXC domain across vertebrates specifically, we obtained the phyloP scores of all nucleotides of the domain; these scores are a measure of conservation across 100 vertebrate species. The resulting distribution of the phyloP scores is shifted towards high values (**Supplementary Figure 2**), again indicating that the CXXC domain is highly conserved. This high conservation is consistent with the importance of the CXXC domain in WDSTS pathogenesis that our analysis of missense variants uncovered.

Motivated by the above results, we also became curious if conservation can also explain why this domain is preferentially enriched compared to the SET domain, which would be the most obvious candidate (given that it is the catalytic domain). It turns out that this is not the case; the two domains are equally conserved (**Supplementary Figure 2**).

We now write in our results:

*“We note here that, while the enrichment of WDSTS-causing MVs within the CXXC domain is consistent with the high conservation of its sequence (****Figure 1D; Supplemental Figure 2****), conservation alone cannot explain the lack of enrichment in the SET domain, since it is as conserved as the CXXC domain (****Supplemental Figure 2****). “*

***“Supplemental Figure 2*** *PhyloP score distribution for the SET and CXXC domain nucleotides of KMT2A.”*

***“Figure 1D:*** *Multiple sequence alignment of the amino-acid sequence of the CXXC domain of KMT2A in eight eukaryotic species. Residues known to be important for DNA binding are marked with red asterisks at the top (see* ***Methods*** *for details). The eight zinc ion-binding cysteines are marked with red asterisks at the bottom.”*

And we now write in our Methods:

*“The amino acid sequence of the CXXC domain of KMT2A orthologs in the eukaryotic species shown in* ***Figure 1D*** *were obtained using the STRING Database [7]. The alignment of the KMT2A orthologous proteins was performed using the Custal Omega multiple sequence alignment tool from EMBL-EBI [8], with default parameters. The phyloP conservation scores of the SET and CXXC domain nucleotides across 100 vertebrates were obtained using the GenomicScores R package [9]."*

Finally, the reviewer’s comment prompted us to examine the phyloP conservation of the CXXC domain, this time grouping nucleotides according to the results from the saturation mutagenesis. Specifically, we grouped the nucleotides based on the number of substitutions that our classifier predicts as having a damaging effect on the protein. This clearly shows that conservation tracks the predicted effects; sites where a greater number of substitutions are predicted as damaging are more conserved. This analysis is an orthogonal validation which supports the accuracy of our variant classification scheme and the results of our saturation mutagenesis, and we therefore added this result as a new panel in main figure 4. We now write in our results:

*“To obtain orthogonal validation for our classifications, we examined the conservation of individual nucleotides coding for the CXXC domain stratified according to the number of substitutions predicted to be damaging, and found strong concordance; sites with a greater number of predicted damaging substitutions are more conserved (****Figure 4C****).”*

***“Figure 4. An ­in silico saturation mutagenesis of the CXXC domain of KMT2A. (C)*** *The distribution of the phyloP score of the nucleotides coding for the CXXC domain, stratified according to the number of substitutions predicted to have a damaging effect (unfolding, compromised DNA binding, stop-gain).*

We have now added the genomic coordinates and transcripts IDs in all supplemental data.

In the methods part the validation of the proposed classification scheme is poorly described. How exactly the training of the rule-based system was performed using the 14 variants for training to obtain the rules shown in Figure 3C? How many splits of the data were performed in hold-out cross-validation? Just one or more? The authors should provide a crosstabulation – a confusion matrix- for validation and for the training data along with the accuracy estimates in Figure 3D. That would help to understand the class balance in the subset of variants used for derivation of the rules.

**Response:** After rereading this methods section, we recognized that it would benefit from a more extensive and clearer description. To address this, we now provide extensive details on how we derived the decision rules that comprise our variant effect classification scheme based on the variants in the training set and on prior information about the contribution of different parts of the amino-acid sequence to the function of the domain (see section “Derivation of the decision rules defining the variant effect classification scheme” below). In addition, we now provide a clear description of how we created our training and test sets. With respect to the training set, we only included variants whose effects on the structure and main function of the domain (i.e. DNA binding) have been experimentally determined. We ensured that we include variants capturing different modes of disruption (unfolding/DNA binding). Within a set of variants corresponding to a given such mode of disruption, we randomly chose a subset to be included in our training set, and we used the rest in our test set; only one random split was performed. In addition, in our test set we included 18 variants seen in patients with phenotypic features of WDSTS [1, 2, 4-6], and 7 and 3 variants from gnomAD and TOPMed [10, 11], respectively, which are expected to be benign. We note here that for the WDSTS variants in the test set we only know that they are pathogenic, but we do not know their precise damaging effect. Therefore, for these variants we are not able to assess whether the precise label that our classifier assigns (unfolding/compromised DNA binding) is true. However, we can still assess the positive predictive value and true positive rate of our classifier by asking if known damaging variants (pathogenic WDSTS variants or variants with an experimentally determined detrimental effect) are correctly classified as causing some type of damage (either unfolding or disruption of DNA binding). To clearly convey this information to the reader, we have now heavily restructured and rewritten the relevant parts of the Methods section, which now stands as follows:

*“****AF2 structure predictions***

*Structure predictions from AF2 were generated using AF2 Colaboratory (v2.0), which does not use templates (which would be expected to bias the mutant structure towards the structure of the wild-type domain [4]). We do not provide a prediction for variants which result in a structure where: a) the pLDDT value of the beta sheets, positioned at the distal ends, drops below 70, or b) the pLDDT value drops to 85 or lower for more than two residues that are not positioned and the distal ends of the domain, or at residues that fall within a secondary structure that is predicted to be absent.*

***Electrostatic potential calculations***

*Secondary structure visualization and computation of mean Coulombic values were performed using UCSF ChimeraX (version 1.2.5) [40], with the AF2 predicted structure as input. MVs were classified as causing a change in electrostatic potential (ESP) if the mean electrostatic potential value of the mutant structure deviated by more than 0.2 (in either direction) from the wild-type domain, whose mean value is equal to 6.28.*

***Derivation of the decision rules defining the variant effect classification scheme***

*We obtained the experimentally determined effects of MVs in the CXXC domain of KMT2A from Allen et al and Cierpicki et al [7]. We then used 14 of these variants to form our training set. These variants were chosen to ensure a representation of different types of disruption (unfolding of the domain/defective DNA binding) within the training set. Within a group of variants causing the same type of disruption, a random subset was selected for inclusion in the training set (4 out of 8 variants causing unfolding, 6 out of 11 variants compromising DNA binding), whereas the rest were included in the test set used for evaluating the performance of our classifier (see section below). Based on the training set, as well as existing biological knowledge about the structure and function of the domain, we derived the decision rules defining our classifier as follows.*

*Our initial decision rule classifies variants which lead to substitutions of the zinc-ion binding cysteine residues as causing unfolding of the domain, since this was the case for all such variants in the training set (C1161A, C1173A, C1189A, D1166A, R1192A). Our next decision rule then classifies variants which lead to substitutions of amino acids responsible for forming direct hydrogen bonds with the DNA (Supplemental Table 2; Cierpicki et al [7]) to amino acids that are incapable of forming hydrogen bonds (Ala, Cys, Gly, Ile, Leu, Met, Phe, Pro, Val) as resulting in compromised DNA binding [41]; this was the case with two such variants in the training set (K1186A and Q1187A) and is consistent with the known chemistry underlying protein-DNA contacts.*

*We subsequently divide the classification scheme into two cascades, based on whether the variant to be classified affects the secondary structure of the domain. For the first cascade, pertaining to variants that do not cause a change in CXXC structure (as determined by AlphaFold2; see previous section), we use the change in ESP (computed using ChimeraX; see previous section) and the position of the variant to determine its effect. There were two variants (N1172A, C1188A) in our training set that were experimentally shown to have no effect on the folding of the domain or on DNA binding. We found that these variants caused neither a change in secondary structure, nor a change in ESP. Therefore, we included a decision rule classifying variants that affect neither the secondary structure nor the ESP as benign. There was also one variant (R1153A) that was experimentally shown to have no effect on folding or DNA binding, which we found caused an ESP change without a concomitant secondary structure change, but the ESP change was at a functionally non-important residue (facing away from the DNA backbone and thus unlikely to impact DNA binding). We thus decided to label variants causing no change in secondary structure and changes in ESP at functionally non-important residues as benign. By contrast, there were two variants (K1176A and R1151A) experimentally shown to compromise DNA binding, which we found caused no change in secondary structure, but caused an ESP change at a functionally important residue (that is, a residue that has been experimentally implicated in electrostatic interaction with the DNA backbone, or a residue forming hydrogen bonds with the DNA, or a residue at the structurally important KFGG site (Supplemental Table 2) [7, 42, 43]). Therefore, we included a decision rule classifying variants which cause no change in secondary structure but cause ESP changes at these sites as resulting in compromised DNA binding.*

*Finally, for the second cascade, variants that demonstrate a change in secondary structure as well as a change in ESP are classified as compromising DNA binding based on the experimentally determined effect of variants R1154A (loss of beta sheets) and D1175A (loss of an alpha helix). Additionally, similar to the prior cascade, we included a decision rule labeling variants that cause a secondary structure change, do not affect ESP, but affect functionally important residues, as resulting in compromised DNA binding, based on prior knowledge about the function of the domain. Finally, variants that cause secondary structure changes but have no impact on ESP and are not positioned at functionally important residues are classified as having no effect.*

***Test set:***

*As described above, we included a random subset of variants from each potential type of disruption (unfolding/compromised DNA binding). In addition, we included 18 variants seen in patients with phenotypic features of WDSTS [14, 17, 18, 20, 21], and 7 and 3 variants from gnomAD and TOPMed [12, 44], respectively, which are expected to be benign. With regards to the WDSTS variants, we know that they are pathogenic, but we do not know their precise damaging effect. Therefore, for these variants we are not able to assess whether the precise label that our classifier assigns (unfolding/compromised DNA binding) is true. However, we can still assess the positive predictive value and true positive rate by asking if known damaging variants (pathogenic WDSTS variants or variants with an experimentally determined detrimental effect) are correctly classified as causing some type of damage (either unfolding or disruption of DNA binding).*

***Saturation mutagenesis:***

*In our saturation mutagenesis, we chose to not provide a prediction for the single variant (R1151P) which causes major steric hindrance in the DNA binding face of the domain, since we do not have data to determine the potential impact of this variant. With respect to the MVs that were classified as disrupting the electrostatic potential, the majority caused changes approximately equal to 0.5, with the minimum being 0.31.”*

Finally, we now also provide a confusion matrix in **Supplementary Figure 6C,** which is now as follows:

***“Supplemental Figure 6.*** *Variant classification scheme from the* ***(A)*** *training set and* ***(B)*** *test set. Variants that do not fall under the correct classification according to the scheme are underlined.* ***(C)*** *Confusion matrix from test set results. “*

What is the rationale of the In-silico saturation mutagenesis of 445 variants? It is not clear whether these variants were created artificially and then classified or they were taken from the existing resources. What is the rational of this analysis, how it is useful for KMT2A variant interpretation?

**Response:** Our motivation for performing the *in silico* saturation mutagenesis was to create a resource for the community (mostly clinical geneticists), by providing a prediction for the effect of all possible variants within the CXXC domain. Clinical geneticists seeking to establish a diagnosis for patients are often confronted with missense variants that have not been seen before; this can make it hard to assess if they are pathogenic or not. Our saturation mutagenesis results can now be used as evidence for or against pathogenicity of a new variant in the CXXC domain. Moreover, our results may be of interest to basic researchers studying the function of the domain. Finally, we believe that, potentially, our approach where we derive an accurate AlphaFold2-based classifier and then use it to perform *in silico* saturation mutagenesis may be applicable to many other disease-relevant proteins; if that turns out to be true, it will help create a vast resource useful for clinicians and basic scientists alike.

Upon rereading the original text, we recognize that our rationale was not clearly conveyed, and we have modified our text which now stands as follows:

*“Clinical geneticists seeking to establish a diagnosis for patients are often confronted with missense variants that have not been seen before; this can make it hard to assess if they are pathogenic or not. The accuracy with which our classifier performs motivated us to perform an in silico saturation mutagenesis for the CXXC domain of KMT2A. Our goal was to create a resource that will enable rapid classification of any newly encountered missense variant in this domain, so that these classifications can be used as supporting evidence in the clinical setting.”*

The ChIP-seq and RNA-seq data were downloaded from paper that uses a mouse model that deletes KMT2A conditionally in adult excitatory forebrain neurons and hippocampal CA. Therefore, it is hard to make firm conclusions around the impact of loss of KMT2A on gene expression in WDSTS as it is a neurodevelopmental disorder and therefore KMT2A may have different targets and effects on chromatin and gene expression during development rather than in the adult brain that has already developed. Conclusions around ChIP-seq and RNA-seq data regarding deposition of histone methylation and gene expression are generalized; authors should address temporal limitation of their mouse model. Additionally, the mouse model used is only KO in neurons. This should also be considered as a limitation as gene expression is different in different brain cell types and loss of KMT2A in these other cell types may also contribute to pathology (either cell autonomously or non- autonomously, meaning loss in other cells impacts neurons as well)

**Response:** We agree with the reviewer about all these caveats, and we have now updated the text in our manuscript to acknowledge these limitations and draw more cautious conclusions. We now write in our Results:

*“Taken together, these results show that, at least in adult excitatory CA hippocampal neurons: a) KMT2A preferentially acts at high-CpG-density regions, and b) its catalytic activity has little effect on gene expression, suggesting that it may also not affect higher-level phenotypes.”*

And in the Discussion, after we discuss our interpretation of the ChIP- and RNA-seq results, we now write:

*“We emphasize here, however, that our findings are based only on data from adult excitatory neurons from the CA region of the hippocampus, whereas gene expression and histone marks are known to be dynamic during development and vary between different cell types. Consequently, future work is needed to assess whether our conclusions generalize to other cell types (neuronal or not) involved in WDSTS pathogenesis, and to earlier developmental stages, when the disease process most likely initiates.”*

The source of the ChIP-seq and RNA-seq data (reference 4) includes ChIP-seq for H3K4me3 along with H3K4me1. Is there a particular reason only the H3K4me1 data was used?

**Response:** We have now performed an analysis of the H3K4me3 ChIP-seq data as well, and have included it in the manuscript. The results mirror those obtained from the H3K4me1 analysis. First, the disruption of H3K4me3 is seen predominantly at CpG-rich locations. Second, we see that changes in H3K4me3 following KMT2A cKO show no systematic relationship with gene expression changes. Finally, we found that the majority of promoters (±1kb of TSS) with H3K4me1 peaks also bore H3K4me3 peaks (81%) (**Supplemental Figure 3B**). When we examined such promoters that have both marks, we observed that the cKO mice had both decreased H3K4me1 signal and decreased H3K4me3 signal (**Figure 2B**). Put together, these data are consistent with a model where KMT2A is normally recruited to CpG-rich promoters, where it is required for deposition of both H3K4me1 and H3K4me3. However, the loss of these marks upon KMT2A loss has no systematic effect on gene expression. These results agree with our domain enrichment analysis which shows that the recruitment of KMT2A at CpG-rich regions is important (since disease-causing missense variants are enriched in the CpG-binding CXXC domain), but not because the histone modifying activity of KMT2A at these regions is important (since disease-causing missense variants are not enriched in the catalytic SET domain). We have now rewritten our Results section, which now reads:

*“Since the CXXC domain mediates binding to clusters of unmethylated CpG dinucleotides [3], our domain enrichment results imply that KMT2A exerts its most important function at CpG-rich regions. Our results also imply that its most important function is not its catalytic activity as a histone methyltransferase, given the lack of MV enrichment in the SET domain. We sought to test these implications, by comparing genome-wide histone methylation (H3K4me1 and H3K4me3) and gene expression patterns in hippocampal CA (Cornu Ammonis) neurons from mice with Kmt2a knockout in excitatory neurons (Kmt2a cKO) as well as wild-type mice (using ChIP-seq and RNA-seq data;* ***Methods****) [18].*

*First, we found a strong relationship between the CpG-richness of a region and the probability of H3K4me1/3 disruption. CpG-rich peaks are much more likely to exhibit disruption upon Kmt2a cKO compared to CpG-poor peaks (****Figure 2A****; 5 wild-type vs 3 cKO mice; p<2.2e-16;* ***Methods****). Reassuringly, there is strong concordance between the changes of the two histone marks in the mutant mice; at the vast majority of promoter regions (+/-1kb from TSS) which bear both H3K4me1 and H3K4me3 peaks, both marks show decreased intensity in the mutant mice (****Figure 2B****;* ***Supplementary Figure 3A****). However, although regions with the strongest evidence for histone methylation disruption (1st p-value decile) most frequently correspond to promoters (39% and 86% for H3K4me1 and H3K4me3, respectively; 3.3-fold and 1.3-fold enrichment compared to regions within the 10th p-value decile, respectively; Fisher’s exact test, p<2.2e-16;* ***Supplemental Figure 3B****), we found no evidence for a systematic impact of promoter H3K4me1 or H3K4me3 disruption on gene expression (5 wild-type vs 6 cKO mice;* ***Methods****). Specifically, genes whose promoters have disrupted H3K4me1 or H3K4me3 are not significantly more likely to be differentially expressed compared to genes without histone methylation disruption at their promoter (****Figure 2C, D****; p = 0.91 and p = 0.13 for H3K4me1 and H3K4me3, respectively, when testing for a shift in p-value distribution between the 1st and 10th deciles with the one tailed Wilcoxon rank-sum test;* ***Methods****). Taken together, these results show that, at least in adult excitatory CA hippocampal neurons: a) KMT2A preferentially acts at high-CpG-density regions, and b) its catalytic activity has little effect on gene expression, suggesting that it may also not affect higher-level phenotypes.”*

Figure 2 has now been updated with this new analysis:

*“****Figure 2****. The relationship between disrupted H3K4me1/3, regional observed-to-expected CpG ratio, and gene expression in Kmt2a-deficient mice. (****A****) The percentage of disrupted H3K4me1 peaks (left) and H3K4me3 peaks (right), stratified based on the observed-to-expected CpG ratio of the underlying peak sequence. (****B****) Scatterplot of the log_2_ fold change of H3K4me1 peaks against the log_2_ fold change of H3K4me3 peaks at promoters (+/-1kb from the TSS) that harbor peaks for both marks. (****C****) The percentage of differentially expressed genes, stratified based on the p-value of associated promoter peaks (+/- 1kb from the TSS) from the differential H3K4me1 analysis (left) and H3K4me3 (right) analysis. (****D)*** *Scatterplot of the log2 fold change of H3K4me1 peaks (left) and H3K4me3 (right) against the log2 fold change of gene expression of the downstream gene. Each point corresponds to a gene-promoter pair. In cases where multiple peaks were present at the same promoter, the average log2 fold change was computed.”*

Supplemental Figure 3 is now:

***“Supplemental Figure 3: (A)*** *Venn diagram depicting the overlap between promoters (+/- 1kb from TSS) harboring H3K4me1 peaks and those harboring H3K4me3 peaks.* ***(B)*** *Genomic annotation of peaks within the 1^st^ (yellow dots) and 10^th^ p-value decile (purple dots) for H3K4me1 peaks (left) and H3K4me3 peaks (right).”*

The author’s focused their RNA-seq analysis on genes in relation to H3K4me1 and find no systematic impact of H3K4me1 disruption on gene expression. As a positive control for the analytical approach used in this study, authors should confirm that their analytical pipeline can first replicate (within reason, expecting some variation due to differences in analytical pipelines) the previous published data (reference 4) which found “471 genes down- and 225 genes upregulated in Kmt2a cKO”?

**Response**: To address the reviewer’s comment we first set out to obtain the list of differentially expressed genes from the Kerimoglu *et al.* paper, but realized that this list is actually not provided. We then reached out to the senior author to request the list, but did not receive a response. Therefore, we are unable to directly demonstrate that we replicate the results of the differential expression analysis of these authors. However, we have now performed quality control checks to evaluate the robustness and reliability of our analysis. These QC checks include:

a) Assessment of the distribution of the resulting p-values (**Supplemental Figure 4A**), which shows a distribution consistent with a two-component mixture; one component corresponding to non-differential genes (p-values distributed uniformly between 0 and 1), and one component corresponding to differential genes (p-values concentrated close to 0). Such a distribution is indicative of a well-calibrated differential expression test.

b) A principal component analysis based on the expression matrix, which shows that the mutants are separate from the wild-type on PCA space (**Supplemental Figure 4B**).

c) An MA plot (**Supplemental Figure 4C**), which indicates no obvious systematic biases in the expression data.

We now write in the relevant methods section:

*“To evaluate the robustness and reliability of our differential expression analysis, we performed the following quality control checks. First, we examined the distribution of the resulting p-values (****Supplemental Figure 4C****), which is consistent with a two-component mixture; one component corresponding to non-differential genes (p-values distributed uniformly between 0 and 1), and one component corresponding to differential genes (p-values concentrated close to 0). Such a distribution indicates a well-calibrated differential expression test, and the existence of true differential hits. Second, we performed a principal component analysis using the expression matrix after applying a regularized log transformation for variance stabilization, as implemented in the rlog() function in DeSeq2; this revealed that the mutants cluster separately from the wild-type on PCA space (****Supplemental figure 4A****). Finally, an MA plot (****Supplemental Figure 4B****) indicates no obvious systematic biases in the expression data.”*

Supplemental Figure 4 is now:

***“Supplemental Figure 4: (A)*** *The histogram of p-values from the differential expression RNAseq analysis.* ***(B)*** *PCA plot based on the expression matrix, after a variance stabilizing transformation (see* ***Methods****).* ***(C)*** *MA plot of the log_2_ fold-change against the mean of normalized counts from the differential expression analysis. Differentially expressed genes are colored in blue. “*

Minor Weaknesses / Corrections

Not clear what Figure 4 represents. It would be clearer if the domain CXXC would be represented as a track in the genome browser with overlaid variant classifications.

**Response**: We have now modified **Figure 4A** to make the representation of our saturation mutagenesis results clearer. Our current visualization is similar to the reviewer’s suggestion, in that we depict the nucleotides horizontally (as they would be depicted on the browser) and we display the variant classifications on top of each nucleotide. We have split the nucleotides into three sets of 50 nucleotides each, so that the figure doesn’t look crammed. We believe the new version of the figure (shown below) is substantially clearer. For instance, one can now immediately locate nucleotides coding for the cysteine residues of the domain, by spotting columns where all three substitutions lead to unfolding of the domain (dark red squares). As another example, nucleotides coding for residues which physically engage the DNA backbone are also easy to locate; these are the columns where most squares are light read, indicating that substitutions compromise DNA binding.

***Figure 4. An ­in silico saturation mutagenesis of the CXXC domain of KMT2A. (A)*** *Heatmap depicting the predicted effect for each nucleotide substitution within the CXXC domain.* ***(B)*** *The percentage of variants with different predicted effects.* ***(C)*** *The phyloP conservation score distribution of the nucleotides of the saturation mutagenesis grouped by how many substitutions were predicted to cause damaging effect.*

It would helpful if the numerical summaries of the features used in the derivation of the classification scheme would be provided along with the training and testing data.

**Response**: We have now included in supplemental table 3 summaries of the features from our training and test set data. This includes the secondary structure change, electrostatic potential change and position of the affected amino acid.

It would be helpful if the proposed scheme would be discussed in view of ACMG variant interpretation guidelines. Which evidence class this method could potentially support?

**Response**: In the ACMG variant interpretation guidelines, predictions from *in silico* tools are used to provide supporting evidence of benign impact (BP4 evidence class), and supporting evidence of pathogenicity (PP3 evidence class). Therefore, these are the evidence classes that our method would support as well. It is worth noting that many of the other computational tools for variant classification rely on similar inputs, such as conservation and population frequency. Our method is to a large extent orthogonal to these other tools, because it is the first method that predicts variant effects by leveraging accurate 3D protein structure prediction by AlphaFold2. Therefore, we expect that the use of our classifier in combination with other tools may prove particularly powerful for predicting the effect of variants in the CXXC domain of KMT2A. We now state this in our discussion, where we write:

*“Within the context of the ACMG variant interpretation guidelines, our classifier can be used as an in silico tool that provides supporting evidence of benign impact (BP4 evidence class) or of pathogenicity (PP3 evidence class). We highlight that it is the first such tool that incorporates accurate 3D protein structure prediction by AlphaFold2, and does not directly use inputs such as conservation and population frequency, which are used by most other tools. Thus, we believe the use of our classifier in combination with other tools may prove particularly powerful for predicting the effect of variants in the CXXC domain of KMT2A.”*

Suggest the addition of a sentence describing AlphFold2 in the introduction or a reference to guide the reader.

**Response**: We have now added a sentence in the introduction where we introduce AlphaFold2 and provide the appropriate reference:

*“By leveraging the recent breakthrough in protein structure prediction from primary amino acid sequence by the deep neural network-based AlphaFold2 [4], we combine accurate 3D structure prediction with existing biological data in order to create an effect classification scheme for missense variants in the CXXC domain.”*

The OMIM abbreviation of the syndrome is WDSTS

**Response**: We have now changed the abbreviation throughout the manuscript to WDSTS to follow the OMIM nomenclature.

From the hold-out test set on the classifier, can you elaborate on the variants that were classified incorrectly? Is there rationale to explain the misclassifications or can you comment on how these misclassified variants may highlight a limitation of this classification scheme?

**Response**: We have now elaborated on these variants in our discussion, where we write:

*“There were three variants in our hold-out test set that were classified erroneously. One of them (from the WDSTS cohort) is in fact annotated as a variant of uncertain significance, suggesting that our classification is not necessarily inaccurate in that case, but rather the “true” label may be incorrect. The other two erroneously classified variants may yield insights into possible limitations of our method. The TOPMed variant misclassified as resulting in compromised DNA binding may reflect the inability of our classifier to place effects along a gradient of severity; in other words, this variant may indeed affect DNA binding as we predict, but only to a mild extent not enough to cause severe disease, and is thus still present among healthy individuals. On the other hand, the pathogenic variant misclassified as benign is located in the structurally important KFGG site in the distal loop of the domain. This raises the possibility that our classifier could be improved by including an earlier decision rule assessing whether a variant affects the KFGG site, as we currently only utilize this information in later steps.”*

The authors should expand on their discussion at lines 187-191 to explain how their results suggest that lack of KMT2A is both “central to WSS pathogenesis” but also that “the phenotype is not mediated via ensuing defects in the deposition of the histone methylation”, in the context of what “alternative mechanisms might be at play”.

**Response:** We have now expanded our discussion on this. We would like to note here, however, that the vast majority of the existing literature on the phenotypic effects of variants in epigenetic regulators focuses on the association between histone modifications and gene expression. While a few alternative possibilities have recently surfaced (as we now mention in our manuscript text), at the moment these are speculative, and very little is known with certainty. We view our results as evidence that more research aimed at elucidating such mechanisms is warranted, and has the potential to contribute to our understanding of WDSTS. We now write in our discussion:

*“However, they also suggest that the phenotype is not mediated via ensuing systematic defects in histone methylation-dependent gene expression, but rather that alternative mechanisms might be at play. Such mechanisms may involve defects in polymerase loading, which has previously been shown to depend on the presence, but not the catalytic activity, of some epigenetic regulators [19].Alternatively, KMT2A may serve as a recruiter of other regulatory factors. Our results emphasize that more research aimed at elucidating such alternative pathways is warranted, and has the potential to contribute to our understanding of WDSTS.”*

The first reference for AlphaFold2 (ref 36) is given at line 289 in the Methods but should be given earlier such as at line 135 in the Results.

**Response**: We now reference AlphaFold2 in our introduction (see also response to comment above), where we write:

*“By leveraging the recent breakthrough in protein structure prediction from primary amino acid sequence by the deep neural network-based AlphaFold2 [4], we combine accurate structure prediction with existing biological data in order to create an effect classification scheme for missense variants in the CXXC domain.”*

Supplemental Table 4, add 2 columns to indicate the nucleotide position and amino acid position for each row/variant. In the text or table legend specify the transcript and protein accession numbers used for the analysis.

**Response**: We have now done this.

The authors mentioned positive and negative prediction values for the classifier. It is confusing and can be misleading as the term “prediction value” is also often used to literally mean the predicted values, like in a regression model for instance. I assume they meant these are true positive and true negative rates (sensitivity and specificity) based on the classifier test results. It would better if they can clarify this.

**Response**: We thank the reviewer for bringing this to our attention. This was a typo; we used the positive predictive value (aka precision; the percentage of variants classified as having a damaging effect that are truly damaging) and negative predictive value (that is, the percentage of variants classified as not any effect on the protein that are truly benign) to assess the performance of the classifier. We have now corrected this in our revised manuscript. In addition, based on the reviewer’s suggestion in a previous comment, we now also provide a confusion matrix (**Supplemental Figure 6,** see also comment above) where we show the true positive and true negative rates of our classifier.

References:

1.	Lebrun, N., et al., *Molecular and cellular issues of KMT2A variants involved in Wiedemann-Steiner syndrome.* Eur J Hum Genet, 2018. **26**(1): p. 107-116.

2.	Baer, S., et al., *Wiedemann-Steiner syndrome as a major cause of syndromic intellectual disability: A study of 33 French cases.* Clin Genet, 2018. **94**(1): p. 141-152.

3.	Miyake, N., et al., *Delineation of clinical features in Wiedemann–Steiner syndrome caused by KMT2A mutations.* Clinical Genetics, 2016. **89**(1): p. 115-119.

4.	Jones, W.D., *Genetic and phenotypic investigations into developmental disorders*, in *Wellcome Trust Sanger Institute*. 2017, University of Cambridge: Newnham College.

5.	Stenson, P.D., et al., *Human Gene Mutation Database (HGMD): 2003 update.* Hum Mutat, 2003. **21**(6): p. 577-81.

6.	Landrum, M.J., et al., *ClinVar: improving access to variant interpretations and supporting evidence.* Nucleic acids research, 2018. **46**(D1): p. D1062-D1067.

7.	Jensen, L.J., et al., *STRING 8--a global view on proteins and their functional interactions in 630 organisms.* Nucleic Acids Res, 2009. **37**(Database issue): p. D412-6.

8.	Madeira, F., et al., *The EMBL-EBI search and sequence analysis tools APIs in 2019.* Nucleic acids research, 2019. **47**(W1): p. W636-W641.

9.	Puigdevall, P. and R. Castelo, *GenomicScores: seamless access to genomewide position-specific scores from R and Bioconductor.* Bioinformatics, 2018. **34**(18): p. 3208-3210.

10.	Karczewski, K.J., et al., *The mutational constraint spectrum quantified from variation in 141,456 humans.* Nature, 2020. **581**(7809): p. 434-443.

11.	Taliun, D., et al., *Sequencing of 53,831 diverse genomes from the NHLBI TOPMed Program.* Nature, 2021. **590**(7845): p. 290-299.

12.	Jumper, J., et al., *Highly accurate protein structure prediction with AlphaFold.* Nature, 2021. **596**(7873): p. 583-589.

13.	Pettersen, E.F., et al., *UCSF ChimeraX: Structure visualization for researchers, educators, and developers.* Protein science : a publication of the Protein Society, 2021. **30**(1): p. 70-82.

14.	Cierpicki, T., et al., *Structure of the MLL CXXC domain-DNA complex and its functional role in MLL-AF9 leukemia.* Nature structural & molecular biology, 2010. **17**(1): p. 62-68.

15.	Hubbard, R.E. and M. Kamran Haider, *Hydrogen Bonds in Proteins: Role and Strength*. 2010.

16.	Frauer, C., et al., *Different Binding Properties and Function of CXXC Zinc Finger Domains in Dnmt1 and Tet1.* PLOS ONE, 2011. **6**(2): p. e16627.

17.	Ayton, P.M., E.H. Chen, and M.L. Cleary, *Binding to nonmethylated CpG DNA is essential for target recognition, transactivation, and myeloid transformation by an MLL oncoprotein.* Molecular and cellular biology, 2004. **24**(23): p. 10470-10478.

18.	Kerimoglu, C., et al., *KMT2A and KMT2B Mediate Memory Function by Affecting Distinct Genomic Regions.* Cell Reports, 2017. **20**(3): p. 538-548.

19.	Dorighi, K.M., et al., *Mll3 and Mll4 Facilitate Enhancer RNA Synthesis and Transcription from Promoters Independently of H3K4 Monomethylation.* Mol Cell, 2017. **66**(4): p. 568-576.e4.
